# Extracting Key Traffic Parameters from UAV Video with On-Board Vehicle Data Validation

**DOI:** 10.3390/s21165620

**Published:** 2021-08-20

**Authors:** Donghui Shan, Tian Lei, Xiaohong Yin, Qin Luo, Lei Gong

**Affiliations:** 1 Research Center of Traffic Safety and Emergency Security Technology, CCCC First Highway Consultants Co., Ltd., Xi’an 715000, China; sdhcjj@126.com; 2 Guangdong Rail Transit Intelligent Operation and Maintenance Technology Development Center, College of Urban Transportation and Logistics, Shenzhen Technology University, Shenzhen 518118, China; yinxiaohong@sztu.edu.cn (X.Y.); luoqin@sztu.edu.cn (Q.L.); gonglei@sztu.edu.cn (L.G.)

**Keywords:** UAV video, traffic information extraction, vehicle detection and tracking, validation experiment, accuracy

## Abstract

The advantages of UAV video in flexibility, traceability, easy-operation, and abundant information make it a popular and powerful aerial tool applied in traffic monitoring in recent years. This paper proposed a systematic approach to detect and track vehicles based on the YOLO v3 model and the deep SORT algorithm for further extracting key traffic parameters. A field experiment was implemented to provide data for model training and validation to ensure the accuracy of the proposed approach. In the experiment, 5400 frame images and 1192 speed points were collected from two test vehicles equipped with high-precision GNSS-RTK and onboard OBD after completion of seven experimental groups with a different height (150 m to 500 m) and operating speed (40 km/h to 90 km/h). The results indicate that the proposed approach exhibits strong robustness and reliability, due to the 90.88% accuracy of object detection and 98.9% precision of tracking vehicle. Moreover, the absolute and relative error of extracted speed falls within ±3 km/h and 2%, respectively. The overall accuracy of the extracted parameters reaches up to 98%.

## 1. Introduction

Traffic monitoring is a fundamental step for intelligent transportation systems (ITS) applications. Obtaining vehicle level traffic data through traffic monitoring is of great significance in the management of transportation systems. Generally, vehicle level traffic data usually comes from two types of sensors: Eulerian (fixed) sensors and Lagrangian (mobile) sensors. The former includes radars [1], inductive loop detectors [2] or magnetometers [3], visible or infrared traffic cameras [4], while the later usually means GPS-based or other techniques supported by probe vehicles [5,6,7]. However, there are some limitations for both fixed and mobile sensors while monitoring traffic state information. Firstly, the range of monitoring is restricted to the distribution of these sensors. The detected traffic information is usually a section data or zone data with limited length. Secondly, the sampling rate of probe vehicles has important influence on the reliability of traffic state evaluation [8]. Therefore, it is sometimes difficult to obtain traffic data at vehicle’s level considering the situation with sparse layout of fixed detectors or limited sampling rate of mobile detectors (such as vehicle trajectory data).

To tackle these issues, an unmanned aerial vehicle (UAV) loaded with a high-resolution camera has been introduced as a promising way of collecting vehicle level real-time traffic information in recent years. As a powerful aerial robot, UAV has been widely explored in the area of geology [9], agriculture [10], hydrology [11], etc. Compared with traditional traffic monitoring approaches, UAV exhibits many advantages including high flexibility, large view range, traceability, simple deployment, low operation cost, and abundant information, etc. [12]. UAV integrates the characteristics of fixed and mobile detectors as it can record the real-time traffic information with different lengths and locations by adjusting flight altitude and path. With the development of deep learning algorithms and image acquisition, UAVs are attracting ever-growing interest in traffic monitoring, especially in extracting vehicle-level data [13]. It has been proven that the accuracy of a static pole-mounted traffic camera ranges from 50% to 75% [14], while the average accuracy of current UAV-based aerial traffic sensing systems is above 80% [15,16].

With these advantages, UAV-driven aerial sensing systems have been widely explored in traffic monitoring for applications such as safety analysis, traffic condition estimation, and driving behavior recognition. For safety-related monitoring, UAVs are used for traffic accident detection [17,18], disaster forecasting or prevention [19,20] and crash risk estimation [21]. Liu et al. (2017) applied image mosaic technology road traffic accident detection using UAV images [17]. Besides safety-related scenario, the most common application of UAV-based imaging system is about traffic condition estimation, such as traffic load monitoring [22], traffic density estimation [23,24], vehicle speed estimation [25], and traffic flow parameters estimation [26]. For driving behavior modelling or recognition, Hao et al. (2019) constructed a relation model to analyze aggressive lane-changing behavior based on an advanced DBN deep learning algorithm through amount of naturalistic on-road experiment using an UAV [27]. Ahmed et al. (2021) utilized a UAV-based geospatial analysis technique for accurate extraction of longitudinal and lateral distances between vehicles to determine the relationship between macroscopic and microscopic parameters of traffic flow [28]. Moreover, UAV-based imaging system is applied for monitoring the environmental impact of traffic in some cases. For instance, Weber et al. (2017) applied octocopter UAV for studying the vertical and horizontal variation of the air pollution plume, which originated from the traffic on a river bridge [29].

The success of UAV-based traffic monitoring system relies on the rapid development of imaging processing techniques. While extracting vehicle-level traffic information through UAV video, a critical step is to detect and track vehicle motion from the aerial video. For years, various algorithms have been applied for vehicle detecting and tracking, such as optical-flow methods [30], feature extraction-matching methods [31], and neural network-based methods [32]. The optical flow method, making use of the correlation between two consecutive image frames, has long been applied in motion detection [33,34]. While, for feature extraction-matching methods, the basic idea is to extract features of the region of interest (ROI) of a same object and matching these features in adjacent images [31]. Feature extraction-matching methods can be divided into photo-based methods [8] and video-based methods [12]. While photo-based methods provide limited information for obtaining dynamic information such as vehicle speed, video-based methods are more preferred in recent researches. Cao et al. (2011) applied a linear SVM classifier for vehicle detection based on feature vectors obtained from a histogram orientation gradient (HOG) feature [35]. Wang et al. (2016) introduced a new vehicle detecting and tracking system which consists of four major modules: image registration, image feature extraction, vehicle shape detection, and vehicle tracking [12].

With the development of computer vision in recent years, multi-object detection models have been proposed to improve the efficient of UAV image data processing. For instance, many neural network frames, such as Fast-RCNN, Faster-RCNN, YOLO, etc., have been developed [36,37,38]. Zhu et al. (2018) developed an advanced deep neural network (DNN)-based vehicle detection methods and applied it to vehicle localization, type (car, bus, and truck) recognition, tracking, and vehicle counting [39]. Another typical applied method, YOLO, which was proposed by Joseph Redmon and others in 2015 [40], is a real-time object detection system based on convolutional neural network (CNN). The most recent version of it is YOLO v3, which has been applied in vehicle detection and is proven to have good detection performance for moving objects [41].

Although various methods have been explored in vehicles in an UAV-monitoring situation, there are still some limitations, such as the relatively low accuracy of vehicle recognition [42]. Previous researches indicate that the accuracy of detecting vehicles in UAV video is lower than 90% [8,42]. Such accuracy may not be enough to provide high-precision data needed for traffic management and control in real-time. Furthermore, drivers’ detailed trajectory information cannot be well obtained [12] considering its performance in vehicle-level traffic analysis, such as a driving behavior study where lane-level vehicle movement should be captured. Moreover, it should be noted that many of the previous studies applied a public data set for model training and validation during vehicle detection and tracking process; less of them conducted field experiment using on-board vehicle device to verify the performance of proposed methods in real-world traffic flow scenario.

This paper therefore attempts to examine the performance of vehicle detection and tracking for key traffic parameter extraction which incorporated real-time on-board vehicle data for validation. In the present work, a novel vehicle detection and tracking approach is developed using YOLO v3 for vehicle detection and deep SORT algorithm for vehicle tracking. Besides, a field experiment is conducted where on-board data from a moving vehicle equipped with high-precision GNSS-RTK is collected to further clarify the performance of proposed model in a real-world scenario. Moreover, two key parameters, vehicle speed and lane position, which are critical in vehicle-level traffic analysis, are extracted to validate the accuracy of the proposed algorithm in a real-world scenario. The structure of this paper is organized as follows: Section 2 gives an introduction about the algorithm used in the present work; Section 3 provides detailed information about the field experiment; and Section 4 focuses on analyzing the results. Finally, conclusions obtained from the present work are summarized in Section 5.

## 2. Traffic Data Extraction Methodology

**Figure 1:**
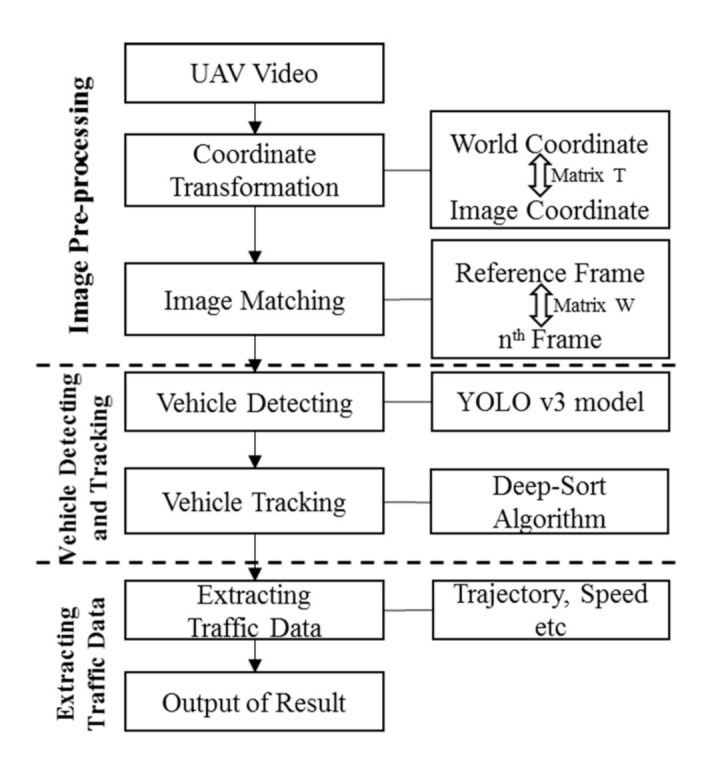
Data extraction procedure from UAV video.

The general procedure of extracting vehicle-level traffic information from UAV video consists of three major steps: image pre-processing, vehicle detecting and tracking, and traffic parameters extraction. In the present work, the proposed approach for extracting traffic data is described in Figure 1. In the image pre-processing step, the uniform coordinate system metrics are determined by the transformation model. Then, a multi-object detection model making use of YOLO v3 and deep SORT is proposed to track vehicle position information over time, based on which critical parameters, such as vehicle trajectory and vehicle speed, are extracted according to the vehicle position information. Methodologies applied are introduced as follows. It should be noted that, in the present work, the overall traffic data extraction process is conducted off-line, as the major purpose of this work is to examine the performance of traffic monitoring when on-board vehicle information is provided. For the implementation, we use open-source Yolov3 with python to realize the proposed method.

### 2.1. Image Pre-Processing

There are two different coordinates for the same target between real-world road and image frames. Theoretically, the image coordinates between different frames should be uniform. However, it is inevitable for the UAV video for a fixed point to slightly move in different frames because of shaking or offsetting. Therefore, image pre-processing is developed to deal with these problems to construct the uniform coordinate and metrics, which consists of coordinate transformation and image matching [43].

(i) Coordinate Transformation: Transformation provides a bridge for a continuous target between real-world and image coordinates. Transformation must select the mark points on the targeted road, such as light, traffic marking, and artificial marker. The coordinate transformation can be realized by developing a congruent relationship for markers in real-world and image coordinate, which is defined as the homographic transformation in the computer vision field [44]. The general model could be expressed in the following equation.

(1) $$\left[\begin{array}{c} u\\ v\\ p \end{array}\right]=T*\left[\begin{array}{c} x_{0}\\ y_{0}\\ z_{0} \end{array}\right]=\left[\begin{array}{ccc} A & D & G\\ B & E & H\\ C & F & I \end{array}\right]*\left[\begin{array}{c} x_{0}\\ y_{0}\\ z_{0} \end{array}\right]$$

where $\left[u\;v\;p\right]$ is the real-world coordinate, and $\left[x_{0}\;y_{0}\;z_{0}\right]$ is the image coordinate of the reference frame. T is the transformation matrix between the real-world and image coordinates. Normally, the altitude of a UAV is higher than 100 m and the corresponding road length is longer than 150 m. Considering the road length and flight height of UAV are much larger than the slope difference, the 3-d problem could be converted into the planar transformation, namely *C*, *F*, and *I* in the matrix *T* is equal to 0, 0, and 1, respectively [45]. As a result, Equation (1) could be expressed in the following:

(2) $$\left[\begin{array}{c} u\\ v\\ 1 \end{array}\right]=T*\left[\begin{array}{c} x_{0}\\ y_{0}\\ 1 \end{array}\right]=\left[\begin{array}{ccc} A & D & G\\ B & E & H\\ 0 & 0 & 1 \end{array}\right]*\left[\begin{array}{c} x_{0}\\ y_{0}\\ 1 \end{array}\right]$$

where A, B, D, E, G, H are the parameters needed to be determined. At least three corresponding mark points are required to determine the matrix *T*. The more mark points there are, the more robust the transformation matrix is. Ideally, the mark points on the road could form the checkerboard shape [46]. The bridge between the real-world and image coordinate could be constructed by the matrix *T* obtained from Equation (2).

(ii) Image matching: Theoretically, a mark point in different image frames follows the same coordinate when the UAV is monitoring the traffic state in a fixed-point hovering way, namely the matrix T is suitable for all of the frames. Moreover, it is inevitable for hovering UAV to slightly move and shake, due to wind or rotation. Therefore, the transformation relationship needs to be determined to unify the coordinate between different frames over time, which is defined as the image matching. The following equation could be used to express the transformation process.

(3) $$\left[\begin{array}{c} x_{n}\\ y_{n}\\ 1 \end{array}\right]=W*\left[\begin{array}{c} x_{0}\\ y_{0}\\ 1 \end{array}\right]=\left[\begin{array}{ccc} a_{1} & a_{2} & a_{3}\\ a_{4} & a_{5} & a_{6}\\ 0 & 0 & 1 \end{array}\right]*\left[\begin{array}{c} x_{0}\\ y_{0}\\ 1 \end{array}\right]$$

where ($x_{0},y_{0}$) is the coordinate of the reference frame $N_{0}$ and ($x_{n},y_{n}$) is the coordinate of the nth frame. W is the transformation matrix, which consists of $a_{1}$ to $a_{6}$ parameters. $a_{1}$ to $a_{6}$ needs to be determined by fixed markers in different images. Consequently, two transformation matrixes (*T* and *W*) develop the relationship and bridge between real-world and image metrics.

### 2.2. Vehicle Detecting and Tracking

After the image pre-processing, the next step is to detect and track vehicles in the region of interest (ROI) by multi-object algorithms. The position information of vehicles over time will be the output in this step, which is the fundamental element to calculate the traffic parameters.

(i) Vehicle detection: In this paper, YOLO v3 is utilized to detect vehicle objects in the frames, which is widely used in the field of object detection due to better efficiency and stability [47]. The most salient feature of YOLO v3 is that it makes detections at three different scales. YOLO is a fully convolutional network and its eventual output is generated by applying a 1 × 1 kernel on a feature map. In YOLO v3, the detection is carried out by applying 1 × 1 detection kernels on feature maps of three different sizes at three different places in the network.

**Figure 2:**
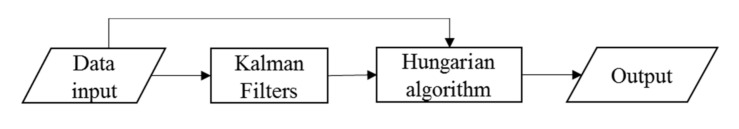
Deep SORT algorithm structure.

The main procedures of YOLO v3 include the following aspects. Firstly, as model input, each image is divided into S × S grid cells. The cell (on the input image) containing the center of the ground truth box of an object is chosen to be the one responsible for predicting the object. Then, each cell predicts the bounding boxes, which contains five critical parameters: center point coordinate ($x_{c}$,$y_{c}$), height (h), width (w), and confidence (*p*). Third, boxes having scores below a threshold (for example, below 0.5) are ignored and non-maximum suppression (NMS) intends to select only one box when several boxes overlap with each other and detect the same object. Finally, the vehicle detection set $N=\left\{N_{i},\;i=1,2,3,\ldots ,n\right\}$ and trajectory set $T=\left\{T_{j},\;j=1,2,3,\ldots ,m\right\}$ are outputted.

(ii) Multiple object tracking: The vehicle information in the frame can be extracted after the implementation of vehicle detection. To uniquely identify a vehicle in different frames until this object leaves the video, the multiple objects tracking algorithm should be used. That is to define the unique ID for the same vehicle in different frames. Considering the possible shadow occlusion problem when vehicles are too close to each other as well as side shadow problems caused by the sunlight, deep SORT algorithm [32] is leveraged to track the objected vehicle in different frames. Deep SORT is the most popular and one of the most widely used multiple objects tracking method, and was proved to have better performance when shadow occlusion problems are considered. The structure of the algorithm is shown in Figure 2.

The procedures of the deep SORT algorithm include the following steps.

Step 1: Input vehicle detection set $N=\left\{N_{i},\;i=1,2,3,\ldots ,n\right\}$ and trajectory set $T=\left\{T_{j},\;j=1,2,3,\ldots ,m\right\}$.

Step 2: Kalman’s prediction. Based on the vehicle history trajectory, the vehicle position set in the nth frame can be predicted, and the predicted set $N_{j}^{n}$ can be obtained

Step 3: Calculate Intersection-over-Union (IoU). $\mathrm{IoU}={\left(x_{ij}\right)}_{n\times m}$, here $x_{ij}$=$\frac{area\left(N_{j}^{n}\right)\cap area\left(N_{j}\right)}{area\left(N_{j}^{n}\right)\cup area\left(N_{j}\right)}$;

Step 4: Associate new detections with the new predictions. The Hungarian algorithm is performed to deal with the association problem.

Step 5: Output the vehicle spatial–temporal data.

Based on vehicle detection and tracking, the coordinate of object vehicles can be output. Naturally, the related parameters, for example, speed, trajectory, etc., can be calculated. The practice needs to measure the accuracy of the proposed method. Therefore, the field experiment is implemented to validate the precision of the proposed approach.

## 3. Experiment

**Figure 3:**
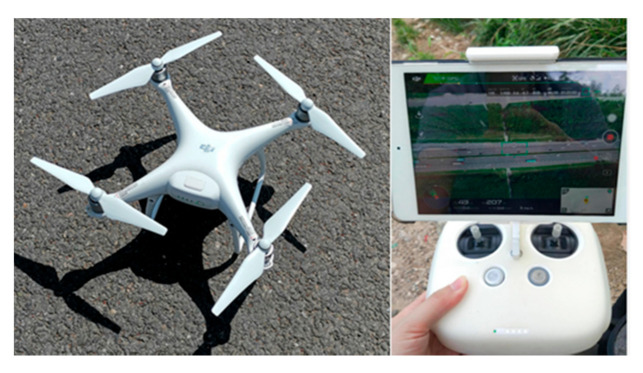
Experimental UAV and control unit.

In the present work, a field experiment was conducted to collect real-world data for modeling training and on-board vehicle data for model validation. The experiment consisted of two major sections: aerial monitoring section and ground monitoring section. In aerial monitoring section, a UAV-imaging monitoring system was set up for collecting video data, consisting of a quadcopter, a camera mount, and a communication module. The DJI Phantom 4 PRO was selected to test the proposed approach in this paper. The core module of UAV is the flight control unit, which is a lightweight multi-rotor control platform. It can be used to adjust the flight height, angle, and hover. The related parameters are shown in Table 1 and Figure 3.

**Figure 4:**
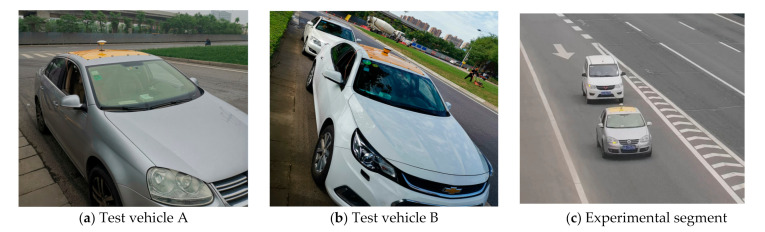
Test vehicles installed with GNSS and OBD.

In Section 2, 2 test vehicles installed with high-precision on-board equipment are selected to collect the reference data needed for validation, as shown in Figure 4. To improve the reliability of experimental validation results, test vehicles were equipped with GNSS for high-precision positioning and an on-board unit (OBD) for collecting other vehicle travelling information. The Trimble SPS985 GNSS was selected to collect high-precision positioning data through the roadside base station, whose precision reached up to centimeter-level. The GNSS frequency was set as 20 Hz to ensure the precision of positioning data. OBD could record real-time vehicle travelling data by reading vehicle CAN and was connected to a cellphone APP through Bluetooth. Besides, OBD platform could record the app data. Therefore, the cross-validation could be realized using data obtained from GNSS and OBD, including speed, acceleration, deceleration, etc. Test vehicles used in the field experiment are shown in Figure 4.

**Figure 5:**
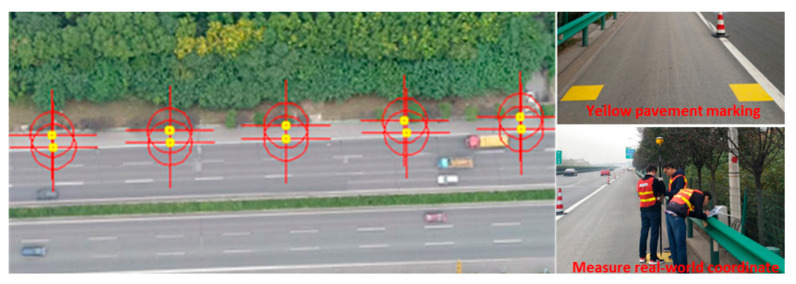
Pavement marking measurements.

The field experiment was implemented on Xi’an Ring Expressway, in Xi’an, China. It should also be noted that the experiment was conducted under good weather conditions. The start location for testing is at the upstream of ZHANG-BA interchange exit and the testing section was about 1 km long. Furthermore, the 50 cm × 50 cm yellow pavement markings were pasted to measure the real-world coordinate at the interval of 50 m, which is a bridge between different coordinates. Totally, there were 386 marked points in the test segment to be measured and collected, shown in Figure 5. It should be noted that yellow pavement markings belonged to one type of road feature points used for solving the coordination transformation problem. While, in non-test situation, other road markings, such as dashed lane lines, road centerline, or deceleration markings, etc., could also be used as road feature points.

**Figure 6:**
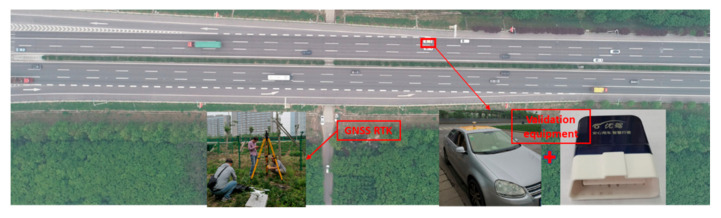
The process of the validation experiment.

**Figure 7:**
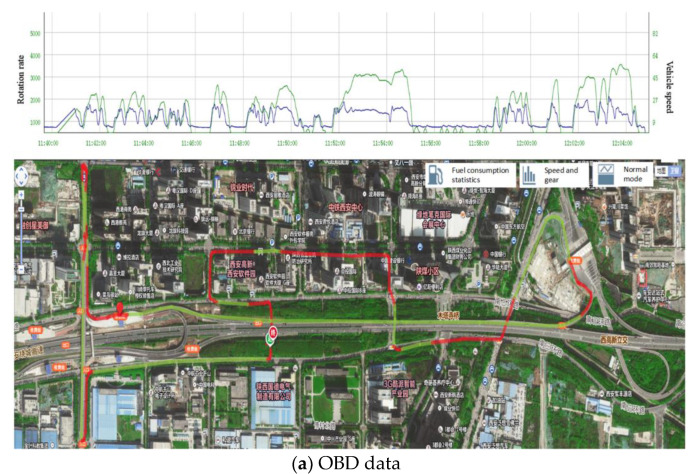

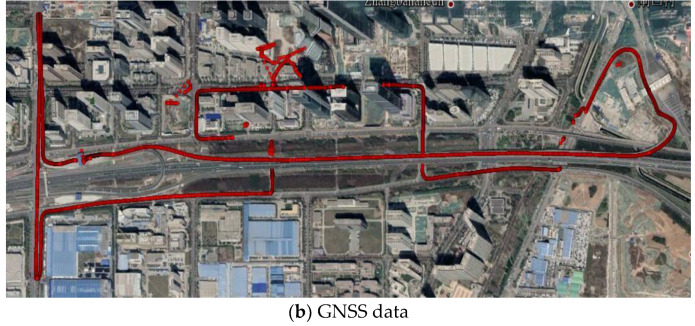
Validation data.

The whole process of the experiment can be presented in Figure 6, including installing the roadside base station, test vehicles, and UAV monitoring. Considering the weather, sunshine, wind, and peak-hour traffic flow, the experiment was chosen at the time from 10:30 am to 5:00 pm. There were seven groups of validation data to be collected at different situations through changing the UAV height and test vehicle speed. The flight height varied from 220 m to 500 m, and the corresponding segment length varied from 150 m to 350 m. The vehicle speed covered 40 km/h to 90 km/h, as shown in Table 2. The UAV height adopted in the present work was relatively higher than existing works. It should be further noted that, during the monitoring process, it was difficult for the test vehicles to always keep a certain driving speed. We then used a speed range as the control variable to test the performance of the proposed system. Each group hovered for 10 min, namely 70 min video data. However, the appearance time of test vehicle in each group experiment was approximately 30 s. A total of 5400 frames containing the test vehicle were produced in this experiment. The real-time data obtained from GNSS-RTK and OBD are shown in Figure 7.

## 4. Results

Based on the abovementioned, the methodology is mainly focused on vehicle detection and tracking. To prove the reliability of the proposed approach, the field experiment was implemented to collect the validation data. Therefore, the precision of vehicle detection, tracking, and parameter extraction is analyzed, respectively.

### 4.1. The Precision of Vehicle Detection

According to the computer vision, precision and recall is the main indicator for evaluating the accuracy of a model or proposed approach [32], which can be calculated as follows.

(4) $$\mathrm{Precision}=TP/\left(TP+FP\right)$$

(5) $$\mathrm{Recall}=TP/\left(TP+FN\right)$$

where *TP*, *FP* represents the true positive and false positive, which refers to the number of the correct detection result for vehicle and the number of the wrong detection for vehicle, respectively. *FN* stands for the false negative, which refers to the number of the wrong detection for non-vehicle. Based on the definition, the average precision (AP) can be calculated.

**Figure 8:**
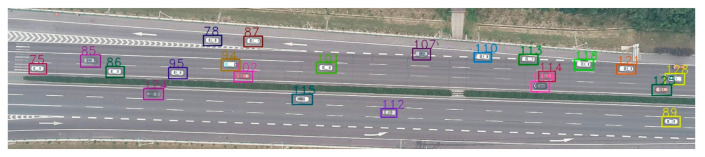
Vehicle detection of UAV video.

The YOLO v3 is trained by setting the different parameters, including momentum, decay, etc. For the model training process, it should be noted that, in order to alleviate overfitting problems, a large number of hand-labeled datasets are applied for the training process to improve the detection accuracy. To obtain better performance, we first adjusted the dataset several times through adding large vehicle samples, increasing samples obtained in scenario with shadow effects, and hand labelling more samples. Then, we adjusted the training parameters, such as the learning rate and other parameters, to improve the detection accuracy of the model. For the entire dataset, we took 80% of them for training and the remaining 20% for testing. Cross validation was not conducted considering the limited training samples and the fact that we did not need to set hyperparameter for the training process. The momentum and decay are equal to 0.93 and 0.005, respectively, in this paper. The initial learning rate is set as 0.0001. For the training process, the loss curve tends to be stable when iteration times reach up to 45,000, but the loss value is more than 0.1. Again, the learning rate is set as 0.00001. The loss curve stabilized again and the loss value is less than 0.1 when iteration times reach up to 85,000. The final learning model is obtained. The results indicate the precision and recall are 98.9% and 99.9%, respectively. The average precision reaches up to 90.88%. The related results are shown in Table 3 and Figure 8.

### 4.2. The Precision of Vehicle Tracking

The multiple object tracking accuracy (MOTA) and multiple object tracking precision (MOTP) are the main indicators for evaluating the performance of object tracking [32]. The following equations can be used to calculate the indicators.

(6) $$\mathrm{MOTA}=1-\frac{\sum_{t}\left(\mathrm{FNt}+\mathrm{FPt}+\mathrm{IDSW}\right)}{\sum_{t}\mathrm{GTt}}$$

(7) $$\mathrm{MOTP}=\frac{\sum_{t,i}{\mathrm{IOU}}_{t,i}}{\sum_{t}\mathrm{Gt}}$$

where FNt refers to the number of the vehicle not associated with an object. FPt represents the number of additional prediction trajectories. ID Switch (IDSW) stands for the number of ID changes. Gt denotes the number of correct associations. MOTA reflects the capacity of tracking object trajectory and is unrelated to position precision. The larger the MOTA, the higher the precision. MOTP mainly measures the precision of object detection, which is independent of an associate degree. The result indicates that the MOTA and MOTP of the proposed approach are 98.9% and 98.8%, respectively, as shown in Table 4.

### 4.3. The Precision of Extracted Speed

**Figure 9:**
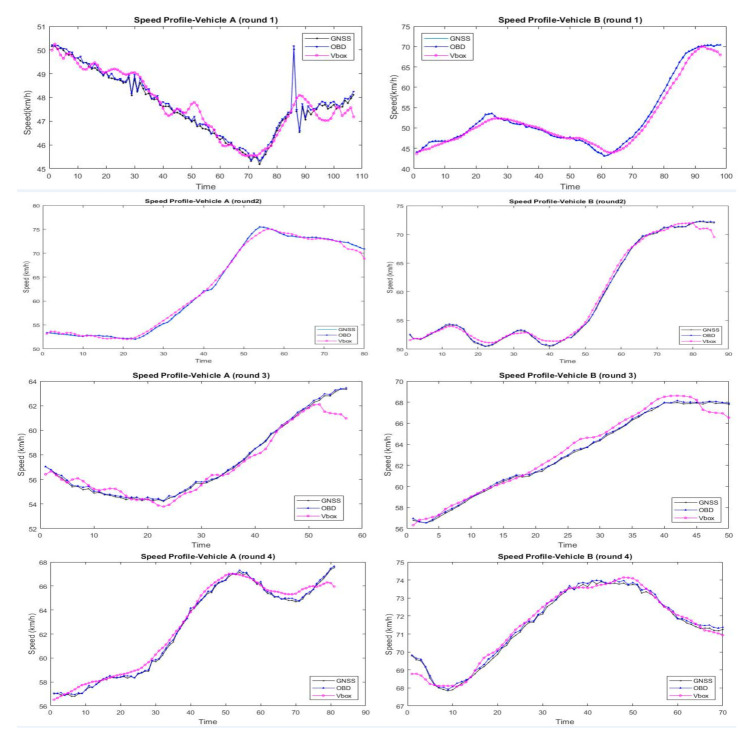

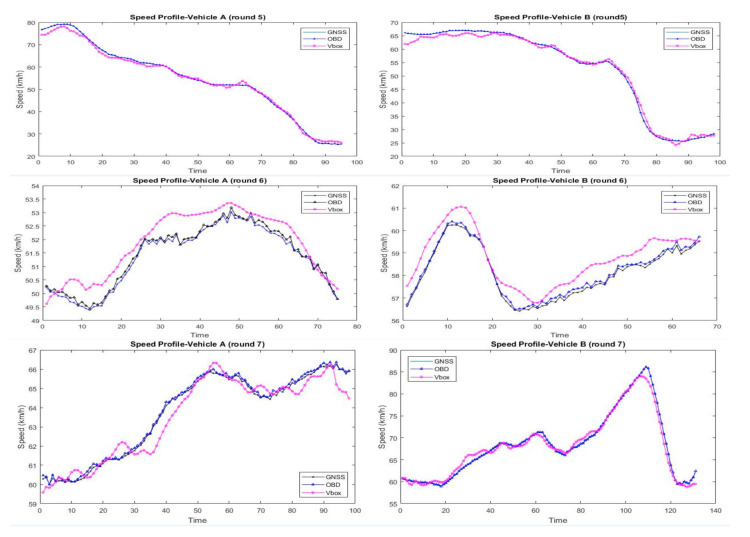
Comparison of speed data.

Based on the vehicle information detected and tracked by the above algorithm, the traffic data, for example, speed, trajectory, driving behavior, etc., can be extracted. Speed is one of the most important parameters for traffic safety. Therefore, it is selected to measure the accuracy of the extracted traffic data by comparing the GNSS and OBD data. Since the OBD data was recorded at the frequency of 5 Hz, while the GNSS was produced at the frequency of 20 Hz, we then interpolated the data obtained from these two sources. For example, there are OBD speed data at 11:00:00 a.m., 11:00:12 a.m., 11:00:24 a.m., 11:00:36 a.m., 11:00:48 a.m., and 11:01:00 a.m. While for GNSS data, the average value of adjacent timestamps (11:00:06 a.m., 11:00:09 a.m., 11:00:12 a.m., and 11:00: 15 a.m.) were taken as the value at timestamp (11:00:12 a.m.). The extracted speed data from the UAV video was integrated similarly. Totally, 1192 speed data were produced to evaluate the accuracy of the proposed approach. The comparison result of seven groups of validation experiments is shown in Figure 9.

As shown in Figure 9, the OBD and GNSS data are almost overlapped, which indicates the high reliability of validation data. The average value of the OBD and GNSS data was produced to be the reference sample. The figures of seven groups of validation experiments show the maximum error of extracted data concentrates on the starting and ending frame, which accounts for the incomplete coverage of test vehicles when it enters and leaves the frame.

The maximum absolute error and relative error were used to measure the accuracy of the sample data. Based on the reference sample, the error of extracted data for test vehicle-A and vehicle-B can be calculated, shown in Table 5. There are 593 and 599 reference data for vehicle-A and vehicle-B, respectively. The results show that the maximum absolute error of vehicle-A and vehicle-B is 2.72 km/h and 2.99 km/h, respectively. The relative error of vehicle-A and vehicle-B is 0.85% and 1.03%, respectively. Based on the above analysis, the results indicate that the precision of the extracted speed reaches up to 98%, the absolute error is less than ±3 km/h, and the relative error falls within 2%. It should be noted that the results described in the present work were obtained under good weather conditions.

## 5. Conclusions

In this paper, the approach for extracting the traffic data from the UAV video was addressed. While UAV-based monitoring exhibits advantages including flexibility, efficiency, large view range, and traceability, this topic has attracted broad attention in the field of traffic monitoring considering the requirements for real-time traffic management and control. This paper proposed an integrated model for vehicle detection and monitoring using YOLO v3 and deep SORT algorithm. While the stability and transformability of the algorithms could be affected by traffic scenario, the situation of taking public dataset for model validation may cause errors in the evaluation of model performance. Therefore, to clarify the feasibility of the proposed approach, field experiment was implemented to validate the accuracy of the extracted data. The GNSS-RTK and OBD were used to record the ground-truth value of test vehicles, namely reference samples. Seven groups of validation experiments were conducted by changing the UAV height and test vehicle speed. The study presents the following findings.

- The accuracy of object detection and tracking of the proposed approach reached up to 90.88% and 98.9%, respectively. Compared with the traditional detection algorithms, vehicle recognition accuracy and robustness was improved.
- The absolute and relative error of extracted speed fell within ±3 km/h and 2%. The overall accuracy of the extracted parameters reached up to 98%. The reference sample obtained from the high-precision equipment (GNSS-RTK and OBD) proves the reliability and feasibility of the proposed approach.
- The proposed approach exhibited strong robustness and reliability. The validation experiments covered the different UAV height (150 m to 500 m) and different test vehicle speed (40 km/h to 90 km/h), but the accuracy of extracted data had little change.

Applying the proposed approach to collect real-time traffic data can provide fundamental elements needed for further traffic analysis and management. Transportation agencies can deploy UAVs to monitor traffic flow and analyze driving behavior, for example, collecting the real-time operating speed to undertake the traffic safety audit. It should be noted that there are still several possible extensions for future work. First, different traffic scenarios (for example, intersection, roundabout, low visibility, etc.) may also be considered to test the robustness of the proposed approach. Second, further analysis about driving behavior could be conducted utilizing real-time traffic data obtained from the present work. Moreover, since the major purpose of the present work is to examine the performance of traffic parameter extraction incorporating real-time on-board vehicle data for validation, the advancement of vehicle detection and vehicle tracking methods is not addressed. Future work should consider more advanced methods.

## Figures and Tables

**Table 1:** Parameters of DJI Phantom 4 PRO.

Title 1	Title 2
Weight	1375 g
Maximum wind speed	10 m/s
Maximum take-off altitude	6000 m
Operative temperature	From 0 °C to 40 °C
Maximum flight time	30 min
Hovering precision	Vertical: ±0.1 m, Horizontal: ±0.3 m
Camera resolution	3840 × 2160 24/25/30p @ 100 Mbps

**Table 2:** Seven groups of the validation experiment.

Number	Appearance Time of Test Vehicle (s)	Test Vehicle Speed (km/h)	UAV Height (m)	The Test Segment Length (m)
1	24	40~70	200	285
2	20	50~80	210	300
3	35	50~70	200	285
4	30	50~80	200	285
5	25	30~80	150	220
6	26	50~60	250	360
7	30	50~90	350	500

**Table 3:** Vehicle detection evaluation.

GT	*TP*	*FP*	*FN*	Recall	Precision	AP
5030	4975	1	55	98.9%	99.9%	90.88%

**Table 4:** Multiple object tracking evaluation.

GTt	FPt	FNt	IDSW	MOTA	MOTP
5090	1	55	0	98.9	98.8

**Table 5:** Result of the validation experiment.

Test Group	Test Vehicle-A				Test Vehicle-B			
	Sample	Average Speed (km/h)	Maximum Absolute Error (km/h)	Maximum Relative Error	Sample	Average Speed (km/h)	Maximum Absolute Error (km/h)	Maximum Relative Error
1	107	47.65	2.33	0.63%	98	52.22	2.99	1.58%
2	80	62.96	1.95	0.67%	86	58.62	2.47	0.80%
3	57	57.29	2.36	0.72%	50	63.02	1.27	0.77%
4	81	62.41	1.56	0.60%	70	71.43	1.01	0.32%
5	95	54.67	2.72	1.64%	98	52.79	2.95	1.55%
6	75	51.33	1.13	1.06%	66	58.15	1.11	1.09%
7	98	63.68	1.42	0.63%	131	68.17	2.93	1.12%
Total	593	57.14	2.72	0.85%	599	60.63	2.99	1.03%

## Data Availability

The data is contained within the article.
